# New Drugs, Appliances, and Things Medical

**Published:** 1891-02-21

**Authors:** 


### NEW DRUGS, APPLIANCES, AND THINGS MEDICAL.

[All preparations, appliances, novelties, etc., of which a notice is desired, should be sent for The Editor, to care of The Manager, 140, Strand, London, W.C.]

### SHEPHERD'S "WARMERS."

Mr. C. W. Shepherd, Pharmaceutical Chemist, Ilkley, has sent us specimens of the above. They consist of curved tins covered with vulcanised rubber. One has a space on the in-curved side for a piece of spongiopiline so that moist applications can be used. The other is plain. The idea is most ingenious. The tins are small enough to be used as muff warmers, for which the plain form is well adapted, or filled with very hot water can be applied to the throat, chest, or a limb, and kept there by means of tapes fastened to the loops which are to be found at each end. The poultice form would be most convenient for any of the affections where a hot, moist application is necessary, besides having the great recommendations of cleanliness, portability, and the power of keeping up a long-continued warmth.

### CAFFYN'S LIQUOR CARNIS.

We have again made analytical examination of this meat preparation and again find it the best we have examined. As we noted before, it consists of the expressed juice of the best parts of English beef, made in Kent. It is preserved by the addition of a harmless and useful carbo-hydrate. It is pleasant to the eye and taste, and contains in a most concentrated form the albuminous constituents of the meat from which it is made. It is not an extract of meat, made at a temperature above 130 degs., F., and it is consequently a valuable food as well as a stimulant. It is the nearest in constituents to raw beef juice, and has the advantage of that form of nutriment, besides being always ready. We have tried it in several cases of acute disease with fever, and found it to answer admirably. More especially is it useful in the dyspepsia of infants with inability to digest milk. A week or ten days' feeding on the Liquor Carnis often puts the intestinal tract of an infant into a healthy condition so that on resuming its normal diet the child continues to thrive. An additional item in its favour would be the absence of the risk of tapeworm infection. This sometimes happens when raw beef juice or scraped meat is given. The method of preparation by the company would obviate this danger. In conclusion we think the company has succeeded in producing a first-rate article, of great value in the sick room in all cases where tissue waste is to be assisted and difficult digestion to be helped on its way.

### BAILEY'S CONCENTRATED CLAM JUICE,

Boston, Mass., U.S.A.

We have all heard of clam chowder, and other succulent dishes in which the American clam appears, and now we can all be tasting it ourselves. Mr. Arthur Bailey has put up in tins a concentrated broth or juice, which can be used as a clam-flavoured basis for the many dishes in which that bivalve appears. On opening a tin, a thin broth appears which is strongly redolent of the clam. To our untutored noses we thought it seemed uncommonly like fresh lobster. However, later on, when we made gastronomic investigation, we perceived the difference. Clam soup and clam chowder, as made with this preparation, are both good, distinctly good. Our chemical investigation showed that the "juice" was wholesomely prepared, and contained a considerable quantity of soluble albumens.

